# Supplementary material for: Isolation and Identification of Bacillus subtilis LY-1 and Its Antifungal and Growth-Promoting Effects
Source: Plants (Basel). 2023 Dec 14;12(24):4158. doi: 10.3390/plants12244158 (PMC10747398; doi:10.3390/plants12244158)
Supplement: Supplementary file 1 [file plants-12-04158-s001.zip › plants-2697964-supplementary.pdf]

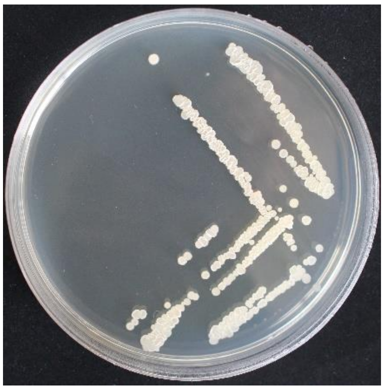

Figure S1 The colony morphology of *Bacillus subtilis* LY-1.

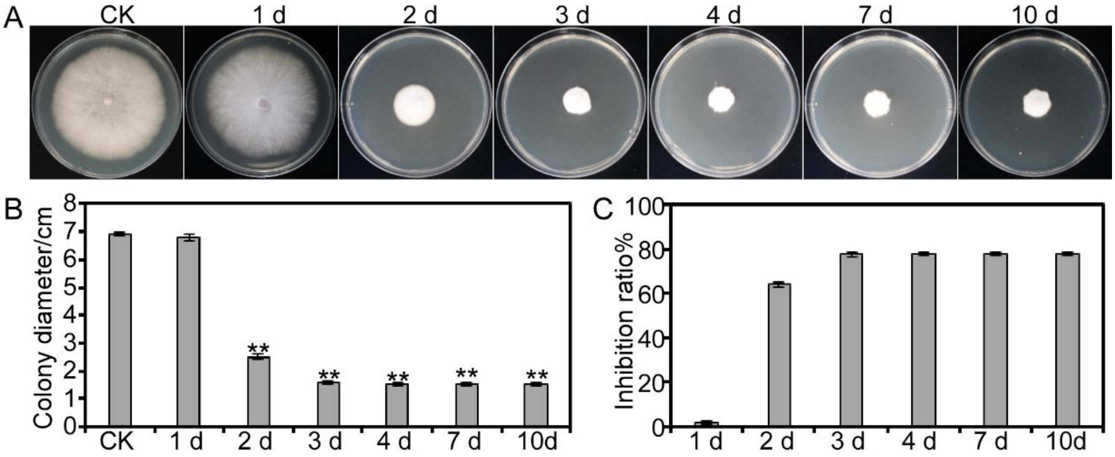

Figure S2 Inhibitory effect of cell-free culture filtrate of *B. subtilis* LY-1 at different times on the growth of *Fusarium* mycelia

A. The growth of *F. oxysporum* mycelia on PDA treated with *B. subtilis* LY-1 cell-free culture filtrate at different times. B. Colony diameter of *F. oxysporum* on PDA treated with cell-free culture filtrate of *B. subtilis* LY-1 at different times. C. Inhibitory effect of cell-free culture filtrate of *B. subtilis* LY-1 at different time on the growth of *F. oxysporum* mycelia.

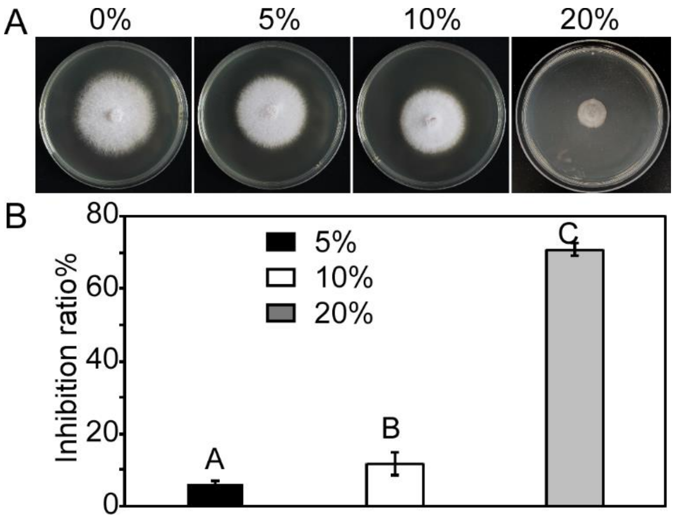

Figure S3 Inhibition of the growth of *F. oxysporum* mycelia by different concentrations of cell-free culture filtrate of *B. subtilis* LY-1

A. The growth of *F. oxysporum* mycelia on PDA treated with cell-free culture filtrate of *B. subtilis* LY-1 at different concentration. B. Inhibitory effect of cell-free culture filtrate of *B. subtilis* LY-1 at different concentration on the growth of *F. oxysporum* mycelia.

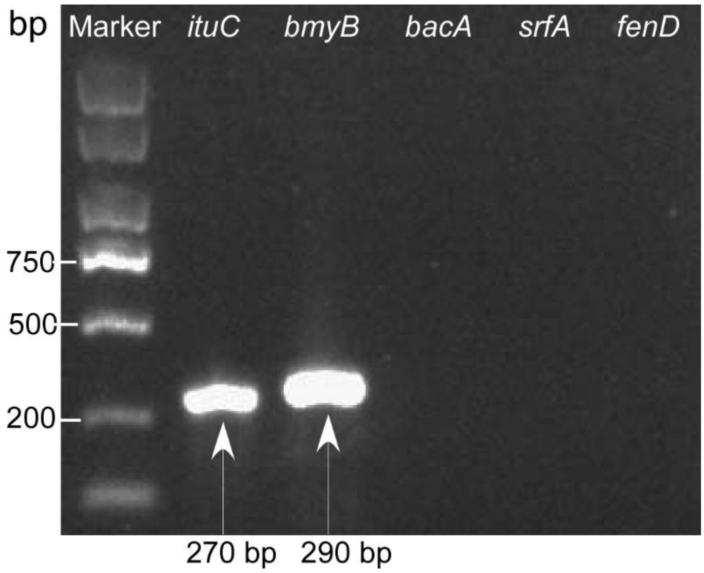

Figure S4 Amplification products of the genes responsible for the synthesis of antimicrobial peptides.

ituC (iturin A synthetase C), *bmyB* (bacillomycin L synthetase B), *bacA* (bacilysin biosynthesis protein BacA), *srfA* (surfactin synthetase subunit 1), *fenD* (fengycin synthetase).

Table S1 Primers for antimicrobial peptide biosynthesis genes.

Name	Sequence	Expected size, bp	Gene product
ituC-F	TGCCATTATTGTCTACGGAG	270	acyl-protein synthetase ItuC
ituC-R	ATAAATCATACAGCCGAC		
bmyB-F	ACGGCAGGTTTTGATTTTT	290	bacillomycin L synthetase BmyB
bmyB-R	CGTTCCTTATCTCCGGA		
bacA-F	CATTTCCAATTTTACTCTTC	410	bacilysin biosynthesis protein BacA
bacA-R	TACTTTTGCCGTGCAAGCTC		
srfA-F	AACGGGGAGCCTGTTCAATA	420	surfactin synthase subunit 1 SrfAA
srfA-R	ACAAGTTCAGGCACCGATTC		
fenD-F	AAAGGTGTGTGGAATTGATG	430	fengycin synthetase FenD
fenD-R	GCTGTCTCCTCTATCAAAAA		
